# Dynamics of a Polymer Network Modeled by a Fractal Cactus

**DOI:** 10.3390/polym10070787

**Published:** 2018-07-18

**Authors:** Aurel Jurjiu, Mircea Galiceanu

**Affiliations:** 1 National Institute for Research and Development of Isotopic and Molecular Technologies, Cluj-Napoca 400293, Romania; 2 Faculty of Physics, Babes-Bolyai University, Street Mihail Kogalniceanu 1, Cluj-Napoca 400084, Romania; 3 Department of Physics, Federal University of Amazonas, Manaus 69077-000, Brazil; mircea@ufam.edu.br

**Keywords:** fractal network, dynamics, rheological quantities, generalized Gaussian structures, scaling

## Abstract

In this paper, we focus on the relaxation dynamics of a polymer network modeled by a fractal cactus. We perform our study in the framework of the generalized Gaussian structure model using both Rouse and Zimm approaches. By performing real-space renormalization transformations, we determine analytically the whole eigenvalue spectrum of the connectivity matrix, thereby rendering possible the analysis of the Rouse-dynamics at very large generations of the structure. The evaluation of the structural and dynamical properties of the fractal network in the Rouse type-approach reveals that they obey scaling and the dynamics is governed by the value of spectral dimension. In the Zimm-type approach, the relaxation quantities show a strong dependence on the strength of the hydrodynamic interaction. For low and medium hydrodynamic interactions, the relaxation quantities do not obey power law behavior, while for slightly larger interactions they do. Under strong hydrodynamic interactions, the storage modulus does not follow power law behavior and the average displacement of the monomer is very low. Remarkably, the theoretical findings with respect to scaling in the intermediate domain of the relaxation quantities are well supported by experimental results from the literature.

## 1. Introduction

In the analysis of polymer dynamics, one of the central questions is in which way the observed complex diffusion patterns and the mechanical response relate to the underlying microscopic geometry. The fact that already simple topological changes, such as the insertion of branch points, leave their trace on dynamical properties of the polymer is well known. The problem of relating the dynamical features of macromolecules with their structure has a long standing history, starting from the landmark works of Rouse [1] and Zimm [2] who focused on the investigation of dilute solutions of linear polymers. With the continuous advancement in polymer synthesis and analysis, the attention turned to macromolecules with more and more complex architectures like star polymers [3,4,5], dendrimers [5,6,7,8,9,10,11,12,13], hyperbranched polymers [5,14,15,16,17,18,19,20,21,22], and polymer networks [23,24,25,26,27,28,29,30]. Nowadays, available experimental techniques in supramolecular chemistry allow for synthesizing a large variety of polymers with precisely controlled molecular structures such as the spherical and cylindrical supramolecular dendrimers [31], the gel-like supramolecular networks [32], and the honeycomb lattices [33], culminating with molecular fractals [34,35,36,37,38].

Fractals, structures with self-repeating patterns at any length scale and a non-integer dimension, are pervasive in nature and emerge in a wide variety of research areas. In physics and chemistry, the fractals are used for describing the dynamics of different polymer networks [39], porous systems [40], stretchable electronics [41], energy storage [42], disordered systems [43], growth phenomena [44], chemical reactions controlled by diffusion [45], and energy transfer [28]. The recourse to the principles of fractal geometry has enabled revealing that most biological elements, either at cellular, tissue or organic level, have self-similar structures within a defined scaling domain which can be characterized by means of the fractal dimension. Nowadays, modern neurosciences recognize the presence of fractal properties in the brain at various levels, i.e., anatomical, functional, pathological, molecular, and epigenetic [46]. Hierarchically organized assembly has been made evident in the formation of protein fibers [47] related to neurodegenerative diseases (Parkinson’s, Alzheimer’s, and Huntington). Applications of fractal measures to pathology and oncology suggest that fractal analysis provides reliable information; fractal analysis helps in discriminating benign from malignant neoplasms [48], and low from high grade tumours [49]. Moreover, fractal analysis of the interface between cancer and normal tissues helps in understanding how cell detachment from the primary mass and infiltration into adjacent tissue occurs through a non-mutational mechanism [50].

Regarding the chemical synthesis of molecular fractals, outstanding results have been reported by Newkome et al. [34] and by Shang et al. [35]. The first results have succeeded the chemical synthesis of the Sierpinski hexagonal fractal, while the second results have reported the achievement of a whole series of molecularly assembled and defect-free Sierpinski triangles, up to the fourth generation. Very recently, the chemical synthesis of the Sierpinski triangles at the fifth generation has been reported by Li et al. [36]. The recent remarkable progress achieved in the design and chemical synthesis of the molecular fractals and the particular relevance of the fractals in many fields of science are solid arguments for further research on the synthesis and analysis of other polymer systems with fractal architectures.

In this work, we analyse the mechanical relaxation dynamics and the structure properties of a fractal polymer network. Its construction stems from that of Sierpinski triangle gaskets. While for the Sierpinski gaskets one uses a building procedure based on face-to-face connectivity of the equilateral triangles, the fractal structure on which we focus is built by using a vertex-type connectivity between the equilateral triangles. The resulting fractal structure has a cactus-like shape and, to a large extent, it can be considered as a structure lying between Husimi cactus [51,52] and the triangular Kagome lattice [53]. Remarkably, the chemical synthesis of the fractal cactus at smaller generations has already been performed [38].

Since we aim to understand the basic features such as the impact of the geometry of the structure on the relaxation dynamics, we prefer to work within the generalized Gaussian structures (GGS) model [5,54,55,56,57,58,59,60], which represents the extension of the Rouse–Zimm chain models [1,2] to polymer systems of arbitrary topologies and which highlight both the connectivity of the molecules under investigation, as well as the influence of hydrodynamic interactions (HI) [2,55]. This leads to a dynamical theory in which the excluded volume effects and the entanglement constrains are neglected. However, in dry polymer networks, the excluded volume effects are often screened, similarly to polymer melts; furthermore, the entanglement constraints should be quite small for sufficiently high cross-link densities and thus short enough network chains. The dynamical quantities on which we focus are the averaged monomer displacement (stretching of the macromolecule under local external forces) and the mechanical relaxation moduli (storage and loss modulus), while, for the structure, we investigate the behavior of the mean-square radius of gyration. These are readily measurable quantities in rheological measurements.

The GGS model offers the possibility to determine, in the Rouse-type approach (the interactions are considered only between nearest neighbour monomers), the full dynamical behavior of the relaxation quantities only by making use of the eigenvalues of the connectivity matrix of the structure. Such great advantage may be lost when dealing with very large structures, hence very large connectivity matrices for which the numerical diagonalizations are practically impossible to perform or are very time-consuming. On the other hand, such hurdles cannot be avoided when precise information about the structure is needed and the numerical diagonalization is the only tool at hand. This is due to the fact that the topological details of the polymer material under investigation are revealed only in the intermediate time/frequency region of the relaxation quantities and this region is always bounded by large crossover domains. Therefore, in order to be able to extract precise information about the structure, its size has to be very large. Remarkably, we avoid the problem by developing an analytical procedure, whereby the whole eigenvalue spectrum of the connectivity matrix is determined iteratively. In the Rouse-type approach, based on the iterative method for obtaining the eigenvalues, we are able to study the relaxation dynamics of the fractal network at very large generations. For instance, the whole eigenvalue spectrum for a fractal network consisting of several hundred millions monomers is obtained in a couple of minutes. The interdisciplinary character of the connectivity matrix is worth commenting on. Examples are provided from many research areas; in graph theory applied to biological systems [61], reaction–diffusion systems [62], in the study of fluorescence depolarization under dipolar quasiresonant energy transfer [28], in the analysis of dynamic processes occurring on networks or inferring many properties related to the networks themself [63,64], for determining the energy levels in Pariser–Parr–Pople (molecular orbital) quantum calculations [65], the dielectric relaxation functions [17], and the NMR relaxation functions [29,66]. Therefore, knowledge of the eigenvalue spectrum is of great importance and leads to interdisciplinary scientific advances. Our goal in the Rouse model is to emphasize that the intermediate time/frequency region of the relaxation quantities is governed by power laws and the main parameter that describes the dynamics is the spectral dimension of the fractal.

The situation changes when one considers the hydrodynamic interactions (HI). These are taken into account in the Zimm fashion by using the preaveraged Oseen tensor [2,55]. In the Zimm-type approach, the dynamical quantities are calculated based on the eigenvalues and the eigenvectors of the product matrix between the connectivity matrix and the hydrodynamic matrix. The Zimm model allows us to solve the eigenvalue problem only numerically, a fact that restricts considerably the possibility to access large size structures. Previous works have shown that, in the Zimm model, the dynamical quantities do not obey power-laws in their intermediate time/frequency domain for fractal networks containing loops on loops (as dual Sierpinski gaskets [39] and Sierpinski hexagon [25]), whereas, for loopless fractals (as Vicsek fractal [17,21]), they do obey power laws. In these works [17,21,25,39], the HI parameter ranged from 0.05 to 0.25. The lost of scaling was attributed to the presence of loops and chiefly loops on loops that produce a position-dependent shortening of the effective bond lengths inside the structure, and thus impair scaling. Our fractal structure contains loops, but not loops on loops; therefore, its study will highlight how the simple loops influence the dynamic behavior of the relaxation quantities under the consideration of the hydrodynamic interactions. Furthermore, we show for our structure that dynamic behavior of the relaxation quantities depends strongly on the value of the HI parameter. The relaxation quantities obey scaling for certain values of the HI parameter, while, for other values, the scaling disappears. The advantage that the small generations of the fractal are already synthesized experimentally, the fact that it contains only loops, the search for scaling in Rouse case, and the dependence of the response functions on the strength of the hydrodynamic interaction in the Zimm case fully justify the choice to investigate this fractal structure.

## 2. Generalized Gaussian Structures

Gaussian models are very valuable because they allow one to study static and dynamic quantities in the framework of linear algebra. The method of choice in this paper is that of the generalized Gaussian structures [5,54,55,56,57,58,59,60], which, as we already mentioned in the Introduction, successfully extended the classical Rouse and Zimm models [1,2] to polymeric systems with more complex architectures. Given that the procedure of GGS was explained in detail in Ref.s [5,54,55,56,57,58,59,60], here we mainly summarize the basic concepts and the main formulas. As a generalized Gaussian structure, we consider an assembly of identical beads connected by harmonic springs with a force constant *K* between topological nearest neighbors. In the framework of the GGS model, the solvent or the surrounding medium is replaced by a continuum, which is felt by the beads through the viscous friction and the stochastic (or random) forces. Here, we consider the homogeneous situation, i.e., each bead experiences the same friction constant ζ with respect to the surrounding viscous medium. The elastic (entropic) springs obey Gaussian statistics. As in the theory for flexible chains, a bead and a spring can be considered as a Kuhn segment [67]. The springs are the representatives of the elastic tensile forces, while the beads play the role of centers on which friction forces apply. The conformation of a polymer is described by the set of position vectors $\left\{R_{i}\right\}$, where $R_{i}\left(t\right)=\left(X_{i}\left(t\right),Y_{i}\left(t\right),Z_{i}\left(t\right)\right)$ denotes the position vector of the *i*th monomer (bead) at time *t*. The GGS assumption is that the potential energy is built only of harmonic terms, involving monomers directly bound to each other; in addition, by including the interactions with external forces $\left\{F_{n}\right\}$, it follows:

(1) $$U\left(\left\{R_{i}\right\}\right)=\frac{K}{2}\sum_{\beta ,m,n}R_{\beta m}A_{nm}R_{\beta n}-\sum_{\beta ,n}F_{\beta n}R_{\beta n}.$$

In the first sum on the right-hand side of Equation (1), all bonds are treated as equal with a thermal equilibrium length *l*. In Equation (1), $K=3T/l^{2}$ is the entropic spring constant, where *T* denotes the temperature in units of the Boltzmann constant $k_{B}$; β runs over the Cartesian coordinates *x*, *y*, and *z*, and the topology of the GGS is accounted by N×N connectivity matrix $A=(A_{ij})$. The connectivity matrix, a discrete version of the Laplacian operator, is constructed according to the following rules: a diagonal element $A_{ii}$ equals the number of bonds emanating from the site *i* and the off-diagonal elements $A_{ij}$ are equal to −1 if the sites *i* and *j* are topological neighbors (i.e., connected by a bond) and zero otherwise. We note that the matrix A is real and symmetric.

We now turn to the interaction mediated by the solvent. First one has, as in all Rouse-type models, a viscous component and there are hydrodynamic couplings between the beads, which depend on the interbead distances. Following Kirkwood and Riseman [68] and Zimm [2], the hydrodynamic couplings between the beads may also be taken into account; one introduces the HI tensor (mobility matrix) $H=(H_{ij})$ [54,55,69] whose components in the preaveraged picture are

(2) $$H_{ij}=\delta_{ij}+\zeta_{r}<l/R_{ij}>\left(1-\delta_{ij}\right).$$

The mobility matrix H is three-dimensional and $R_{ij}=|R_{ij}\left|=\right|R_{i}-R_{j}|$ represents the mutual separation (interbead distance) between the centers of the beads *i* and *j*. Furthermore, the strength of hydrodynamic interactions can be expressed in terms of an effective hydrodynamic interaction radius *a* $\zeta_{r}=\zeta /6\pi \eta_{0}l=a/l$, where ζ is the friction coefficient and $\eta_{0}$ the solvent’s viscosity. Moreover, taking Gaussian distributed interbead distances, one has

(3) $$\langle R_{ij}^{-1}\rangle ={\left(\frac{6}{\pi <R_{ij}^{2}>}\right)}^{1/2}.$$

The polymer is subjected to random forces $f_{i}\left(t\right)$ with a zero average, $\langle f_{i}\left(t\right)\rangle =0$, and the force autocorrelation function is given by $\langle f_{i}\left(t\right)f_{j}\left(t^{\prime }\right)\rangle =2k_{B}T\zeta H_{i,j}^{-1}\delta \left(t-t^{\prime }\right)$, where $H^{-1}$ is the inverse of the matrix $H=(H_{ij})$ and $\delta (t-t^{\prime })$ is the Dirac delta function. It is now a relatively straightforward matter to compute the dynamical properties, since the GGS problem is linear and the different components ($X_{i},Y_{i},Z_{i}$) decouple. With coordinates $Y={\left(Y_{1},Y_{2},\cdots ,Y_{N}\right)}^{T}$ and forces $f={\left(f_{1},f_{2},\cdots ,f_{N}\right)}^{T}$, the corresponding Langevin equation reads in matrix notation [54,55]

(4) $$\frac{\partial Y(t)}{\partial t}+\sigma HAY\left(t\right)=\frac{1}{\zeta }H\left[f\left(t\right)+F\left(t\right)\right],$$

with σ=K/ζ being the inverse of the monomeric relaxation time. Equation (4) has the following formal solution:

(5) $$Y\left(t\right)=\frac{1}{\zeta }\int_{-\infty }^{t}dt^{\prime }exp\left[-\sigma \left(t-t^{\prime }\right)\mathrm{HA}\right]H[f\left(t^{\prime }\right)+\left[F\left(t^{\prime }\right)\right],$$

as can be verified by differentiating the right-hand side with respect to *t*. In order to bring Equation (5) to a more convenient computational form, one proceeds by diagonalizing the product matrix HA, i.e., by determining *N* linearly independent eigenvectors $Q_{i}$ of HA, so that ${\mathrm{HAQ}}_{i}=\lambda_{i}Q_{i}$, where $\lambda_{i}$ are the eigenvalues of HA. For a completely connected structure and for a physically reasonable inclusion of the HI (i.e., the stability of the Zimm approach [70]), the inverse ${\mathrm{HA}}^{-1}$ exists, and the matrix HA has only one vanishing eigenvalue which we denote by $\lambda_{1}$, the other eigenvalues all being positive [71]. Equation (5), representing the motion of individual monomers in external fields, an idealized case of micromanipulation experiments [72,73], can be further simplified. We let the external force start to act at t=0 on one monomer contained in the GGS; then, we average over all possibilities of choosing this monomer randomly. The resulting (quenched) ensemble doubly averaged <<Y(t)>> (averaged over the thermal forces and over all monomers in GGS) reads:

(6) $$<<Y\left(t\right)>>=\frac{F{\bar{H}}_{11}t}{N\zeta }+\frac{F}{\sigma N\zeta }\sum_{i=2}^{N}\frac{1-\exp(-\sigma \lambda_{i}t)}{\lambda_{i}}{\bar{H}}_{ii},$$

where ${\bar{H}}_{ii}=\sum_{k,l}Q_{ik}^{-1}H_{kl}Q_{li}$, so that ${\bar{H}}_{11}$ is simply ${\bar{H}}_{11}=\sqrt{N}\sum_{k}Q_{1k}^{-1}H_{k1}$. It is noteworthy that Equation (6) contains only the eigenvalues, $\lambda_{i}$, of the product matrix HA and its eigenvectors through the elements ${\bar{H}}_{ii}$. In the Rouse-type approach, which neglects the hydrodynamic interactions, the hydrodynamic matrix reduces to the unitary matrix, H=I, i.e., $H_{ij}=\delta_{ij}$ for all *i* and *j*, leading to further simplification of averaged monomer displacement form:

(7) $$<<Y\left(t\right)>>=\frac{Ft}{N\zeta }+\frac{F}{\sigma N\zeta }\sum_{i=2}^{N}\frac{1-\exp(-\sigma \lambda_{i}t)}{\lambda_{i}}.$$

From Equation (7), we remark that, in the Rouse model, for determining the motion of individual monomers in external fields, we need only the eigenvalues of the connectivity matrix A. In addition, we note that, in Equation (7), due to $\lambda_{1}=0$, the motion of the center of mass has separated automatically from the remaining sum. From Equations (6) and (7), the behavior of the motion for extremely short and for very long times is obvious: in the limit of very short times <<Y(t)>>=Ft/ζ, while, for very long periods, one has $<<Y\left(t\right)>>=F{\bar{H}}_{11}t/N\zeta$. Physically, this means that, at very short times, only one bead is moving, whereas, for very long periods, the whole structure drifts. These very general features make clear that the particular structure of the GGS is revealed in the intermediate time domain [5,17,18,21], depending on the whole eigenvalue spectrum of the connectivity matrix A (for Rouse model) or of HA (for Zimm model).

An experimentally readily accessible quantity is the complex dynamic modulus, $G^{*}\left(\omega \right)$, which is usually determined by applying an external harmonic strain to the system. Even more familiar are the storage $G^{\prime }\left(\omega \right)$ and the loss $G^{\prime \prime }\left(\omega \right)$ moduli, which represent the real and the imaginary components of $G^{*}\left(\omega \right)$. For very dilute solutions and for ω>0, the storage and loss mechanical moduli are given by (see also Equations 4.159 and 4.160 of Ref. [55]):

(8) $$G^{\prime }\left(\omega \right)=\frac{C}{N}\sum_{i=2}^{N}\frac{\omega^{2}}{\omega^{2}+{\left(2\sigma \lambda_{i}\right)}^{2}}$$

and

(9) $$G^{\prime \prime }\left(\omega \right)=\frac{C}{N}\sum_{i=2}^{N}\frac{2\sigma \omega \lambda_{i}}{\omega^{2}+{\left(2\sigma \lambda_{i}\right)}^{2}}.$$

For very dilute solutions, one has $C=\nu k_{B}T$, with ν being the number of polymer segments (beads) per unit volume. In Equations (8) and (9), ω represents the frequency and $\lambda_{i}$ are the eigenvalues of the connectivity matrix A in the Rouse-type approach and of the matrix HA in the Zimm-type approach, respectively. In addition, for concentrate solutions (when the entanglement effects are negligible), Equations (8) and (9) are still valid, the only change being in the value of the constant *C* [74]. The factor 2 arises from the second moment of the displacements involved in computing the stress [55]. These relaxation quantities are intimately related to other dynamic quantities, such as the dielectric and the magnetic relaxation.

A basic structural feature of a polymer is its radius of gyration. In the framework of the GGS model, the mean-square radius of gyration depends only on the eigenvalue spectrum of the connectivity matrix [54]

(10) $$R_{g}^{2}=\frac{\langle l^{2}\rangle }{N}\sum_{i=2}^{N}\frac{1}{\lambda_{i}}.$$

## 3. The Fractal Cactus Network and the Eigenvalue Spectrum

**Figure 1:**
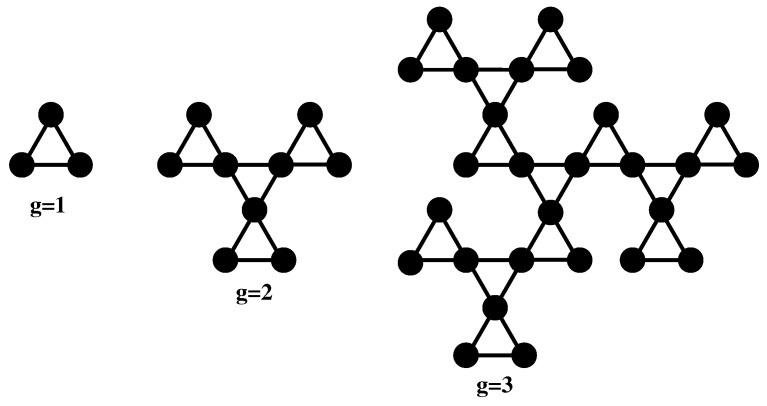
The fractal cactus polymer network at generations 1, 2, and 3.

The general topology on which we focus is displayed in Figure 1, which shows schematically in 2D the first three generations of the cactus-like fractal. As in the case of Sierpinski gasket, the first generation is a simple equilateral triangle. Using a vertex-type connectivity, a new triangle emerges from all the nodes of this central triangle, thus forming the object at second generation. The third generation of the fractal, shown on the right-hand side of Figure 1, is built by binding with an equilateral triangle three identical objects of the second generation. As a general rule, the fractal cactus at any generation, say *g*, is built by linking in regular manner with a triangle three identical fractal objects of generation g−1. Hence, the fractal structure at generation *g* will consist of $N=3^{g}$ beads (monomers). Note that the regular pattern of Figure 1 (embedded in the 2D-Euclidean space) has the same geometric fractal dimension as the Sierpinski triangles [39], namely:

(11) $$d_{f}=\frac{ln\;3}{ln\;2}=1.58496\ldots ,$$

since each generation creates three self-similar objects and the ratio between lengths of the objects from successive generations can be approximated to 2 (for large generations). However, since we deal with a Gaussian model, a much more useful measure will be the Gaussian fractal dimension, $d_{fg}$. The $d_{fg}$ of the polymeric fractal, defined by the size-mass scaling, $R_{g}^{d_{fg}}∼N$, is given by [75,76]

(12) $$d_{fg}=\frac{2d_{s}}{2-d_{s}},$$

where $d_{s}$ is the spectral dimension of the fractal structure. The spectral dimension is an intrinsic parameter, which is determined only by the connectivity of the monomers.

As we already mentioned in the Introduction, real structures with the shape of our fractal cactus already exist; the first three generations of the present fractal cactus have been experimentally synthesized [38]. They are the branched [4] triangulane and the branched [*n*] triangulanes (BTs). Furthermore, the fractal cactus can be seen as a very regular structure interpolating between two non-fractal structures, the triangular Kagome lattice [53] and Husimi cactus (the dual structure of the perfect dendrimer) [51,52], being closer to the latter. Moreover, if each small triangle is replaced with a bead and then each such bead is connected with springs to its nearest neighbors, the resulted hyperbranched loopless structure is very similar to that of the recently synthesized internally functionalized dendrimers [77]. These new dendrimers contain groups that are protected from reaction, so that they do not branch out further.

In what follows, we outline the iterative procedure that allows one to obtain the whole eigenvalue spectrum of the fractal cactus at any desired generation. The detailed calculations are given in Appendix A. The determination of the eigenvalues, i.e., the solution of

(13) (A−λI)Φ=0,

starts from realizing that the structure of the fractal consists of two types of beads: four-coordinated beads and two-coordinated beads; hence, each of the beads of fractal cactus has either four or two nearest neighbors. In the following, we write explicitly Equation (13) for each type of bead and we denote the components of the eigenvector Φ by $\phi_{j}$. For any four-coordinated bead, the eigenvalue equation reads

(14) $$\left(4-\lambda \right)\phi_{i}=\sum_{j=1}^{4}\phi_{j},$$

where $\phi_{i}$ is the eigenvector component of the four-coordinated bead and $\phi_{j}$s are the eigenvector components of its nearest neighbors, which may be either four-coordinated or two-coordinated. For any two-coordinated bead, one has

(15) $$\left(2-\lambda \right)\phi_{k}=\phi_{m}+\phi_{n},$$

where $\phi_{k}$ is the eigenvector component of the double-coordinated bead and $\phi_{m}$ and $\phi_{n}$ are the eigenvector components of its nearest neighbors, one being two-coordinated and the other four-coordinated.

We can now use two specific real-space renormalization transformations to reduce the fractal cactus from generation *g* to generation g−1. These transformations are presented in detail in Appendix A. The result of these is that, in the decimated structure (i.e., the structure at generation g−1 that was obtained by the reduction of the structure of generation *g* through the real-space transformations), Equations (14) and (15) get replaced by (see Equations (A32) and (A14))

(16) $$\left[4-P\left(\lambda \right)\right]\psi_{i}=\sum_{j=1}^{4}\psi_{j}$$

and

(17) $$\left[2-P\left(\lambda \right)\right]\psi_{k}=\psi_{m}+\psi_{n},$$

where $\psi_{i}$, $\psi_{j}$, $\psi_{k}$, $\psi_{m}$, and $\psi_{n}$ are the eigenvector components in the decimated structure and the polynomial P(λ) is given by

(18) $$P\left(\lambda \right)=-\lambda^{2}+6\lambda .$$

As can be seen from Appendix A, each eigenvector component from Equations (16) and (17) is, in fact, a sum of three eigenvector components coresponding to either four-coordinated or two-coordinated beads of the structure before decimation. The iterative procedure, which is based on the fact that the fractal cactus rescales under the real-space renormalization transformations, can now be iterated *k* times, by which P(λ) gets replaced by $p_{k}\left(\lambda \right)=P\left(p_{k-1}\left(\lambda \right)\right)$. For finite fractal cactus structures, this iteration permits (except for the vanishing eigenvalue $\lambda_{1}=0$) determining the eigenvalues at generation *g* from those at generation g−1 through the relation:

(19) $$P\left(\lambda_{i}^{\left(g\right)}\right)=\lambda_{i}^{\left(g-1\right)}.$$

Now, by simply solving Equation (19) with P(λ) given by Equation (18), one finds the relationship between the eigenvalues belonging to successive generations

(20) $$\lambda_{\pm }^{\left(g\right)}=\frac{6\pm \sqrt{36-4\cdot \lambda^{\left(g-1\right)}}}{2}.$$

Evidently, in this way, each eigenvalue of generation g−1 gives rise to two new ones at generation *g*. This procedure also makes it clear that the new eigenvalues keep the degeneracy of their predecessors.

At any generation *g* of the fractal cactus, the whole eigenvalue spectrum of its connectivity matrix is determined as follows: a part of the eigenvalue spectrum is calculated from the eigenvalues of generation g−1 by employing Equation (20); these eigenvalues are complemented by the nondegenerate vanishing eigenvalue $\lambda_{1}=0$ and by $\Delta_{g}$ degenerate eigenvalues equal to 3 each, where the degeneracy, $\Delta_{g}$, is given by

(21) $$\Delta_{g}=1+3^{g-1}.$$

At the first generation, the eigenvalue spectrum consists of vanishing eigenvalue λ=0 and of the eigenvalue λ=3, which is two times degenerate. We call this eigenvalue principal eigenvalue ($\lambda_{p}=3$) because all the others are obtained from it based on Equation (20). It is worth mentioning that the eigenvalue spectrum of the fractal cactus consists of only persistent eigenvalues, and the term persistent eigenvalue means an eigenvalue that appeared at one generation and continues to appear in all subsequent generations. In addition, all eigenvalues of the spectrum are degenerate, except the eigenvalue $\lambda_{1}=0$. It is now a simple exercise to prove that the above outlined procedure gives the whole eigenvalue spectrum. The total number of eigenvalues at generation *g* is

(22) $$N=\sum_{i=0}^{g-1}2^{i}\cdot \Delta_{g-i}=3^{g}.$$

Focusing on the spectral region of small eigenvalues allows one to determine the so-called spectral dimension $d_{s}$ of the fractal cactus. The starting point is the observation that, in the limit of very small λs, one can linearize Equation (18) and obtain as an iteration scheme from Equation (19)

(23) $$6\lambda_{g+1}-\lambda_{g}=0.$$

Let $\rho_{g}\left(\lambda \right)$ be the density of modes at *g*th stage. Since the number of eigenmodes in a given interval dλ of the *g*th stage is compressed into an interval $d\lambda^{\prime }$ of the (g+1)th stage, we must have

(24) $$\rho_{g}\left(\lambda \right)d\lambda =3\cdot \rho_{g+1}\left(\lambda^{\prime }\right)d\lambda^{\prime }.$$

Denoting $\lambda /\lambda^{\prime }=k$ and using the relation for the density of modes $\rho_{g}\left(\lambda \right)∼\lambda^{\frac{d_{s}}{2}-1}$, we get

(25) $$d_{s}=\frac{2\;ln\;3}{ln\;k}.$$

From Equation (23) we get $k=\frac{\lambda_{g}}{\lambda_{g+1}}=6$ and then inserting this value into Equation (25) we obtain

(26) $$d_{s}=\frac{2\;ln\;3}{ln\;6}=1.226294\ldots$$

Inserting the value of the spectral dimension given by Equation (26) into Equation (12) we get the Gaussian fractal dimension, $d_{fg}=3.169925$.

**Figure 2:**
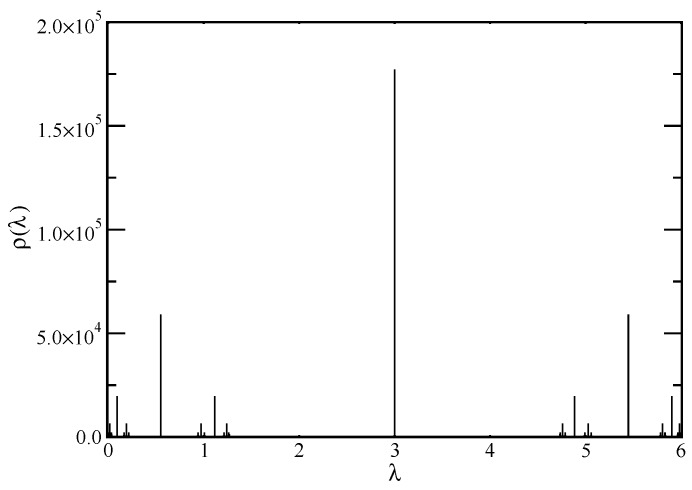
Histogram of the eigenvalues of the connectivity matrix A for the fractal cactus network of size $N=3^{12}$. The width of the bins is 0.001.

A graphical representation of the eigenvalue spectrum of the connectivity matrix, A, which we have determined through the iterative method is displayed in Figure 2. The eigenvalue spectrum of the fractal cactus at generation g=12 (i.e., N=531441) is shown as a histogram, the number of eigenvalues in intervals of width dλ=0.001. The spectrum is limited between 0 and 6. Particularly striking from the figure is that fractal cactus does not have a smooth eigenvalue spectrum; it is highly discontinuous with a multitude of forbidden bands and inherent symmetries.This type of spectrum is multifractal. Nonetheless, as will become clear in the next section, such type of spectrum gives rise to rather smooth relaxation patterns, which scale according to relations based on the spectral dimension.

## 4. Relaxation Patterns

### 4.1. Results Obtained in the Rouse Approach

In this subsection, we will make use of the eigenvalues determined by iterative means in order to calculate the different quantities introduced in Section 2. The first quantity on which we focus is the mean-square radius of gyration, $R_{g}^{2}$. This represents a measure of the size of the polymer, and, in the GGS model, it is expressed by Equation (10). In what follows, we derive an alternative exact analytical expression for the mean square-radius of gyration. Based on the recursive relation Equation (19), the sum from Equation (10) can be evaluated analytically. Equation (19) written explicitly for generation *g* is:

(27) $${\left(\lambda_{i}^{\left(g\right)}\right)}^{2}-6\lambda_{i}^{\left(g\right)}+\lambda_{i}^{\left(g-1\right)}=0.$$

According to the Vieta’s formulas, the two roots (i.e., $\lambda_{i,1}^{g}$ and $\lambda_{i,2}^{g}$) of Equation (27) satisfy the following two relations: $\lambda_{i,1}^{g}+\lambda_{i,2}^{g}=6$ and $\lambda_{i,1}^{g}\cdot \lambda_{i,2}^{g}=\lambda_{i}^{g-1}$; thus,

(28) $$\frac{1}{\lambda_{i,1}^{g}}+\frac{1}{\lambda_{i,2}^{g}}=\frac{6}{\lambda_{i}^{g-1}}.$$

The above procedure is iterated g−1 times backwards until one reaches the principal eigenvalue, $\lambda_{p}$, and the sum of the reciprocal of all non-zero eigenvalues becomes:

(29) $$\sum_{i=2}^{N}\frac{1}{\lambda_{i}}=\frac{1}{\lambda_{p}}\left[\left(\frac{8}{15}\right)\cdot 6^{g}-3^{g-1}-\frac{1}{5}\right].$$

Now, the mean-square radius of gyration at generation *g* is given by:

(30) $$R_{g}^{2}=\frac{1}{N}\left[\left(\frac{8}{15}\right)\cdot 6^{g}-3^{g-2}-\frac{1}{15}\right].$$

This expression is very useful because it can be evaluated only knowing the size of the fractal network. We also remark that the sum of the reciprocal of all non-zero eigenvalues (Equation (29)) is intimately related to the mean-first-passage time of a random walk [16] that represents the expected time to hit a target node for the first time for a walker starting from a source node.

Equations (10) and (30) give identical values of the $R_{g}^{2}$. Besides its simplicity, the advantage of expression (30) is that it allows for large fractal networks (g≫1) to display the scaling behavior of $R_{g}^{2}$ in an analytical form. For large fractal networks, the first term from Equation (30) brings the largest contribution to $R_{g}^{2}$ and we cancel the second and the third terms. Recalling $N=3^{g}$, we have $6^{g}=N^{\ln6/\ln3}$ that enables one to write

(31) $$R_{g}^{2}=\frac{8}{15}N^{ln6/ln3-1}.$$

By setting $c_{1}=8/15$ and relating the exponent with the spectral dimension from Equation (26), the mean-square radius of gyration has the following the power-law form:

(32) $$R_{g}^{2}=c_{1}\cdot N^{\frac{2-d_{s}}{d_{s}}}.$$

**Figure 3:**
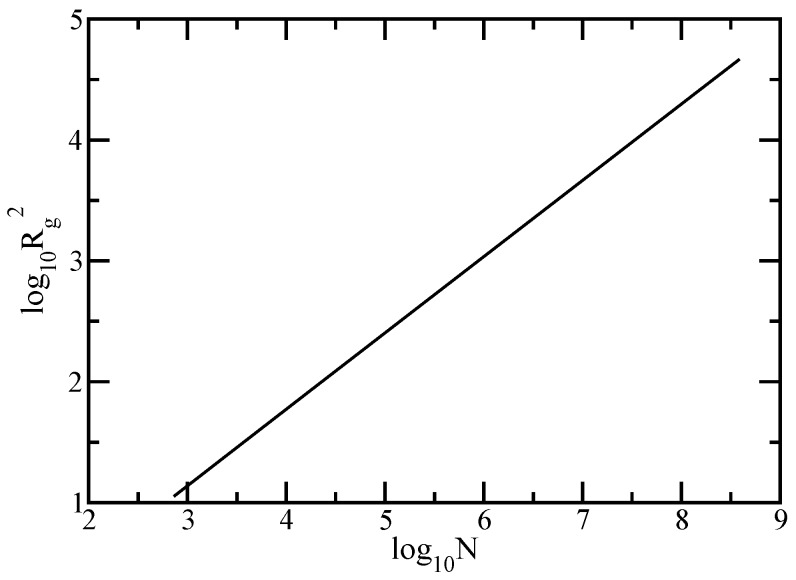
The mean-squared radius of gyration of the fractal cactus network calculated in the Rouse model.

In order to check the validity of the scaling relation predicted by Equation (32), we plot in Figure 3 the results obtained for the mean-square radius of gyration given by Equation (10) and evaluated for fractal cactus structures whose sizes extend from $N=3^{6}$ to $N=3^{18}$. In this model, all bonds have the same length equal to one, thus the mean squared bond length $\langle l^{2}\rangle$ = 1. In the double logarithmic scales of Figure 3, the mean-square radius of gyration appears as a straight line, thus obeying a power-law, $R_{g}^{2}∼N^{\theta }$. The best approximation to our data leads to θ=0.631, the value being very close to the prediction of $\left(2-d_{s}\right)/d_{s}=0.63093$. The achieved results confirm the validity of the derived formula for the radius of gyration.

**Figure 4:**
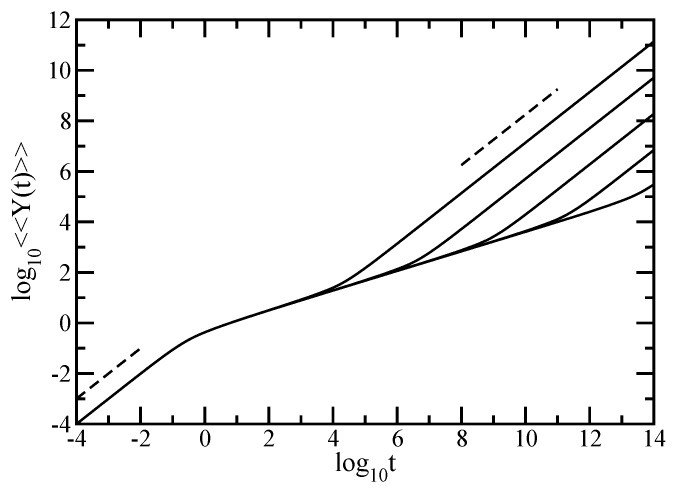
The averaged monomer displacement under the action of an external force in the Rouse model. Displayed in dimensionless units is the <<Y(t)>> for the fractal cactus networks with $N=3^{6}$, $3^{9}$, $3^{12}$, $3^{15}$, and $3^{18}$ from above. For guidance, the black dashed lines indicate that the slope equals 1.

We now turn to the dynamics of individual monomers in external field and evaluate the averaged monomer displacement, a quantity which may be accessed experimentally through micromechanical manipulations [72,73]. Figure 4 displays the results obtained for averaged monomer displacement, <<Y(t)>>, calculated based on Equation (7) in which we set σ=1 and F/ζ=1. The scales of the figure are double logarithmic to basis 10 and the displayed results have been achieved for fractal cactus networks with generations ranging from g=6 to g=18; consequently, the total number of beads in the structure varies from $N=3^{6}$ to $N=3^{18}$. What appears immediately in the double logarithmic scales of Figure 4 is the limiting time behavior; at very short times, <<Y(t)>>∼t meaning that the monomer does not feel any constraints that arise from the connection to the neighboring monomers, while, for very long periods, one reaches the domain where <<Y(t)>>∼t/N, which, in the absence of an external field (based on the Einstein relation for GGS [5,34,59,71]) is the hallmark of simple diffusion. As a guide to the eye the dashed black lines indicate the slope 1. Due to the *N*-dependence of <<Y(t)>> in Equation (7), the curves belonging to fractals of different sizes are shifted with respect to each other. Typical for the topological details of the structure under investigation is the intermediate time domain. Given the double logarithmic scales of the Figure 4, this subdiffusive regime appears as a straight line, a fact which denotes scaling; it obeys $<<Y\left(t\right)>>∼t^{\gamma }$, with, as we proceed to show, $\gamma =1-\frac{d_{s}}{2}$. It is now a simple matter to determine numerically the power-law exponent γ, which is nothing else than the slope of the curves in the intermediate region. Thus, in the intermediate times region, we find for the largest fractal considered, namely for $N=3^{18}$, the exponent γ=0.389. This value should be compared with the theoretical $\gamma =1-\frac{d_{s}}{2}=0.38685$. This very good agreement is due to the fact that the value of *N* is very large; we were able to attain it due to our iterative method for calculation of the eigenvalues.

**Figure 5:**
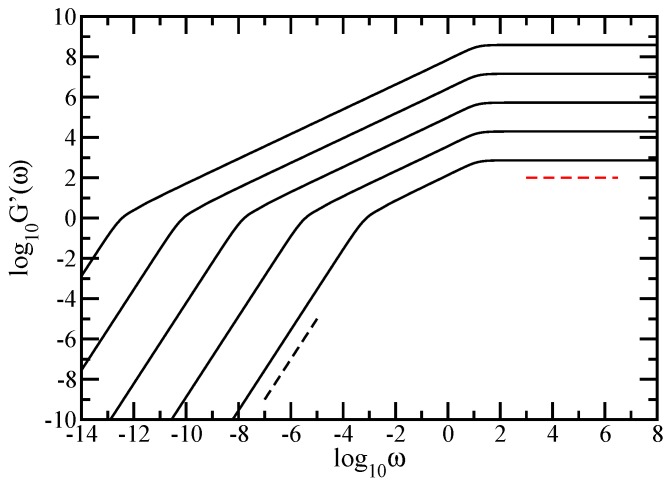
Storage modulus $G^{\prime }\left(\omega \right)$, displayed in dimensionless units for the fractal cactus networks with $N=3^{6}$, $3^{9}$, $3^{12}$, $3^{15}$, and $3^{18}$ from below. For guidance, the dashed black line indicates the slope 2 and the red dashed line indicates the slope 0, Rouse model.

**Figure 6:**
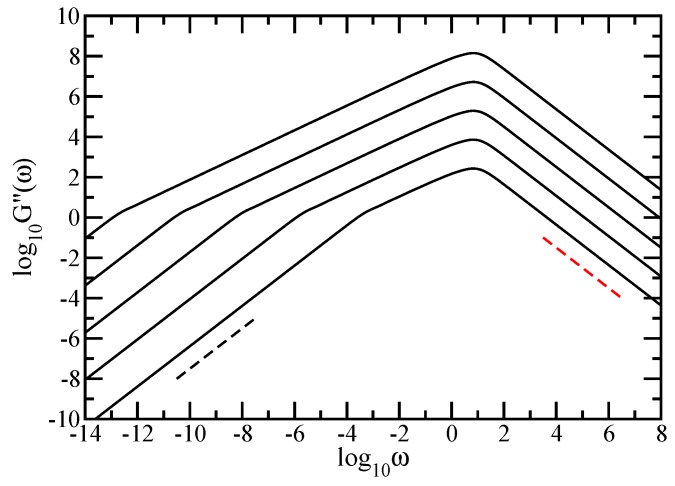
Loss modulus $G^{\prime \prime }\left(\omega \right)$, displayed in dimensionless units for the fractal cactus networks with $N=3^{6}$, $3^{9}$, $3^{12}$, $3^{15}$, and $3^{18}$ from below. For guidance, the dashed black line indicates the slope 1 and the red dashed line indicates the slope −1, Rouse model.

Most measurements on polymers, however, are not monitored in time but in the frequency domain. Given the importance of the rheological measurements on polymers, we continue by focusing on the mechanical moduli, $G^{\prime }\left(\omega \right)$ and $G^{\prime \prime }\left(\omega \right)$, given by Equations (8) and (9), which we present in Figure 5 and Figure 6. In both figures, we have used finite fractal objects ranging from $N=3^{6}$ to $N=3^{18}$. In Figure 5 and Figure 6, we plot Equations (8) and (9) in dimensionless units, by setting σ=1 and C/N=1. The scales in both figures are double logarithmic to basis 10. Evidently from these figures are the limiting, connectivity-independent behaviors at very small and very large frequencies; for ω≪1, one has $G^{\prime }\left(\omega \right)∼\omega^{2}$ and $G^{\prime \prime }\left(\omega \right)∼\omega$, whereas, for ω≫1, one finds $G^{\prime }\left(\omega \right)∼\omega^{0}$ and $G^{\prime \prime }\left(\omega \right)∼\omega^{-1}$. As a guide to the eye, two reference (dashed) lines are plotted at the lower and at the upper limit; in Figure 5, the dashed black line indicates the slope 2 and the red dashed line indicates the slope 0, while, in Figure 6, the dashed black line indicates the slope 1 and the red dashed line indicates the slope −1. Our main focus is again the intermediate frequency region. In both figures, for each fractal object considered, this in-between region appears as a straight line that indicates power-law behavior. Going from $N=3^{6}$ to $N=3^{18}$, we have a change in the minimal slope from 0.662 to 0.614 for $G^{\prime }\left(\omega \right)$, (in Figure 5) and from 0.575 to 0.605 for $G^{\prime \prime }\left(\omega \right)$, (in Figure 6), respectively. The values of the slope obtained for the largest fractal considered should be compared to $d_{s}/2=0.61315$. The agreement is very good. From the accuracy attained here and also from Figure 4, we draw the conclusion that the sole fractal parameter of importance for the relaxation dynamics in the Rouse-type approach is the spectral dimension, $d_{s}$. We also note that the accuracy obtained for $G^{\prime }\left(\omega \right)$ is better than the other one obtained for $G^{\prime \prime }\left(\omega \right)$. This is because the loss modulus is in general a less sensitive measure of the relaxation than the storage modulus. Once again, we remark that, if *N* is small ($3^{6}$ monomers, for instance), due to the substantial crossover domains, the slope which is inferred from the moduli is rather far from $d_{s}/2$; to obtain this value, accurately large structures are needed.

### 4.2. Comparison with Experimental Results from the Literature

To our best knowledge, there is no available experimental data for the averaged monomer displacement and mechanical relaxation moduli of triangulane [38] structures in order to compare with our theoretical results. Remarkably, our theoretical findings in the Rouse model are well supported by mechanical experiments performed on different types of polymers. The comparison with experimental results is done with respect to scaling in the intermediate frequency domain of the relaxation quantities. The authors of Ref. [78] reported power-law behavior in the intermediate frequency domain of the mechanical moduli for the thermoreversible gelation of poly(methyl methacrylate) containing 80% syndiotactic triads (sPMMA) and block copolymers of the MXM type, where M is sPMMA and X is either PBD, hydrogenated PBD (PEB) or poly(styrene-b-butadiene-b-styrene) (SBS) triblock. For these types of gels, the authors reported values of the exponent ranging from 0.65 to 0.7. The smallest value is closer to our theoretical values 0.614 (for $G^{\prime }\left(\omega \right)$) and 0.605 (for $G^{\prime \prime }\left(\omega \right)$) obtained for the largest fractal considered in Figure 5 and Figure 6. In Ref. [79], the authors studied the influence of different hydrogen-bonding side groups on the dynamical behavior of functional poly(n-butyl acrylate) melts and cross-linked networks. One of their samples, the (AP15), has the value of the exponent 0.6 for $G^{\prime }\left(\omega \right),$ which is similar to our theoretical value. The authors of Ref. [80] studied the structure formation and the rheological properties of series of telechelic polyisobutylenes, functionalized with hydrogen-bonding end groups. They have reported that the behavior of mechanical relaxation moduli of such supramolecular gels follow a power-law with the value of the exponent of 0.58 which is also close to our theoretical value. The theoretical results are also in very good accordance with the results reported in Ref. [81] for the anomalous self-diffusion in associative protein hydrogels. The obtained scaling exponent for the frequency dependences of the relaxation moduli has the value 0.62. In Ref. [82], the authors investigated the physical gelation in living polymer networks. They used the aqueous solution of poly(vinyl alcohol) (PVA) and sodium tetraborate decahydrate (borax) as a model material. The obtained slopes in the intermediate frequency region of the relaxation moduli of such networks was 0.59, also in good agreement with our values.

Our theoretical results obtained for the average motion of individual monomer in the Rouse model are also supported by the experiments. Again, the comparison with the experimental results is made with respect to scaling in the subdiffusive time regime. In Figure 4, for the largest fractal considered, we obtained in the intermediate time domain of <<Y(t)>> a slope of 0.389. This theoretical value is in good accordance with 0.42, the exponent of the mean square displacement determined in Ref. [83] for solutions of water-soluble polyethylene oxide (PEO) with high molecular weight.

### 4.3. Results Obtained in the Zimm Approach

The fact that the hydrodynamic interactions strongly influence the dynamics of dilute polymer solutions is well known. While in the Rouse approach a monomer interacts only with its nearest neighbors, with the hydrodynamic interactions present, the velocity of a monomer affects all the other monomers through the flow of the solvent. As we already stressed in Section 2, the hydrodynamics interactions are taken into account by using the preaveraged Oseen tensor [2,55]. In the Zimm model, preaveraging the forces between the beads may constitute a serious approximation, the stability of the Zimm approach depends on the strength of the hydrodynamic interaction. The preaveraged scheme may even lead for large interaction parameters $\zeta_{r}$ to unphysical behaviors, such as the appearance of negative eigenvalues, negative diffusion coefficients and related instabilities. For values of the hydrodynamic interaction parameter below the instability of the Zimm approach, preaveraging is in general reasonable and leads to qualitatively correct results.

The relaxation dynamics of some regular fractal structures has been intensively investigated in several previous studies [17,21,25,39]. For the dual Sierpinski gaskets and for the Sierpinski hexagon, the conclusion drawn was that the relevant physical quantities that describe the dynamics (averaged monomer displacement and mechanical moduli) do not obey power laws in the Zimm approaches. For these types of fractals, the loops appear at all scales and they lead to a position-dependent shortening of the effective bond lengths inside the structure, and thus impair scaling. Instead, for the Vicsek fractals which do not present loops, it has been reported [17,21] that the dynamical quantities do scale in the Zimm-type approach. In these studies, the HI strength varied from 0.05 to 0.25.

**Figure 7:**
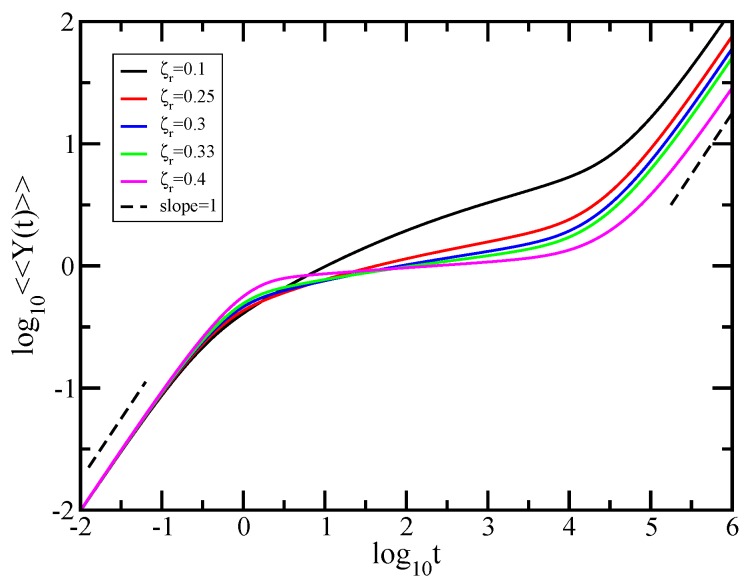
Averaged monomer displacement <<Y(t)>> of the fractal cactus network under the action of external forces in the Zimm model. Displayed in dimensionless units are the results for the network size $N=3^{8}$ and hydrodynamic interaction strength $\zeta_{r}=0.1$, 0.25, 0.3, 0.33, and 0.4. For guidance, the black dashed lines indicate that the slope equals 1.

In what follows, we examine relaxation dynamics of the fractal cactus when HI interactions are active. Again, we start by focussing on the averaged displacement <<Y(t)>>, given by Equation (6) in which we set σ=1 and F/ζ=1. Parallelling Figure 4 for the Rouse case, the HI-results are presented in Figure 7 for the finite fractal cactus network with size $N=3^{8}$ and for $\zeta_{r}$ ranging from 0.1 to 0.4. Note that all employed values of $\zeta_{r}$ are below the instability of the Zimm approach, which for the present fractal starts from $\zeta_{r}^{*}=0.5$. From this value, in the eigenvalue spectrum of the product matrix, negative eigenvalues start to appear. The results obtained for each value of the HI strength are indicated as follows: black solid line ($\zeta_{r}=0.1$), red solid line ($\zeta_{r}=0.25$), blue solid line ($\zeta_{r}=0.3$), green solid line ($\zeta_{r}=0.33$), and magenta solid line ($\zeta_{r}=0.4$). As before, for very short times, all curves merge; this is the domain where <<Y(t)>>=Ft/ζ. Furthermore, for long periods, one reaches the domain $<<Y\left(t\right)>>≃F{\bar{H}}_{11}t/N\zeta$. In the logarithmic scales of Figure 7, these two domains appear as straight lines with slope 1. The two dashed reference lines exhibit the that slope equals 1. In addition, we find that, under HI, the intermediate time range gets smaller (as was also established in several previous works [17,21,25,39]). As discussed before, typical for the structure under investigation is the intermediate regime. In this time domain, no scaling is evident for the curves corresponding to $\zeta_{r}=0.1$ and 0.25. These curves are not smooth, and they display a concave curvature in the double logarithmic plot. Such non-scaling behavior is in line with that obtained for the dual Sierpinski gaskets and also for the Sierpinski hexagon under the same values of the HI strength [25,39]. Surprisingly, the increasing of the hydrodynamic interaction parameter will completely change the behavior of the averaged monomer displacement. In the double logarithmic scales of the figure, the curves achieved for $\zeta_{r}=0.3$ and 0.33 appear as straight lines, thus obeying power law. Linear fits in the intermediate time ranges of these curves lead to slopes of 0.12 for $\zeta_{r}=0.3$ and 0.1 for $\zeta_{r}=0.33$. By further increasing the hydrodynamic interaction strength, the slope of the curve in the intermediate time domain tends to zero. We have obtained for $\zeta_{r}=0.4$ a slope of 0.04. Under strong hydrodynamic interactions, the displacement of the monomer is very small in the intermediate time domain. The physical meaning is that, under strong HI, it is not sufficient to pull only one monomer in order to unfold the structure.

**Figure 8:**
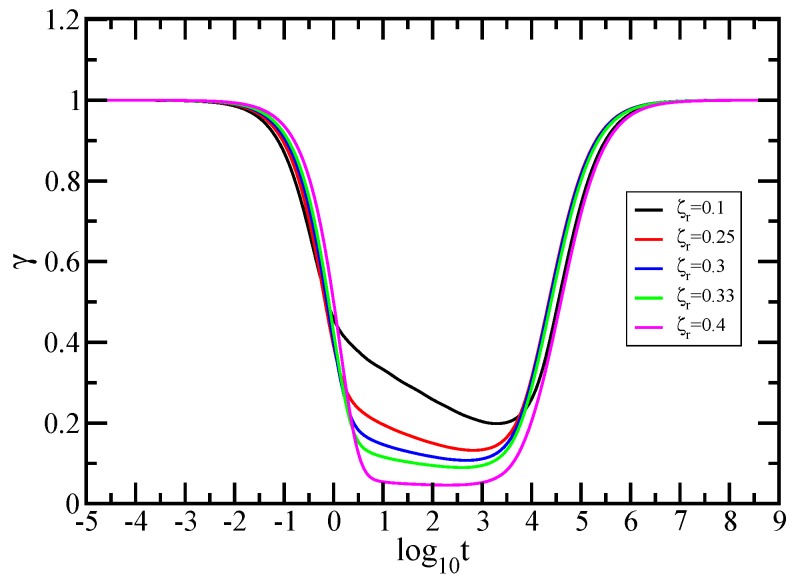
Local slopes γ of the curves of Figure 7. The inset gives the considered values of the hydrodynamic interaction strength. Zimm model.

In order to display a more quantitative analysis and for a better visualization of the dynamical behavior, we plot in Figure 8 the derivatives (i.e., local slopes), γ=d(ln<<Y(t)>>)/d(lnt), of the curves of Figure 7. To avoid confusion, the derivatives are displayed with the same colors (see inset) as their correspondents in Figure 7. We note that in the figure the *x*-axis is logarithmic and the *y*-axis is linear. The limiting cases with slope 1 are evident. For the values of $\zeta_{r}=0.1$ and 0.25 (the black and red solid lines), one can clearly observe that there is no plateau in the intermediate time region, a fact which indicates unequivocally that there is no scaling. The situation changes vastly with increasing the hydrodynamic interaction strength. For the values of $\zeta_{r}=0.3$ and 0.33 (the blue and green solid lines), one can see clearly in the intermediate time domain the appearance of a plateau that indicates power law behavior. For $\zeta_{r}=0.4$, the plateau value is around 0.04, meaning that the monomer in this time domain moves very little.

**Figure 9:**
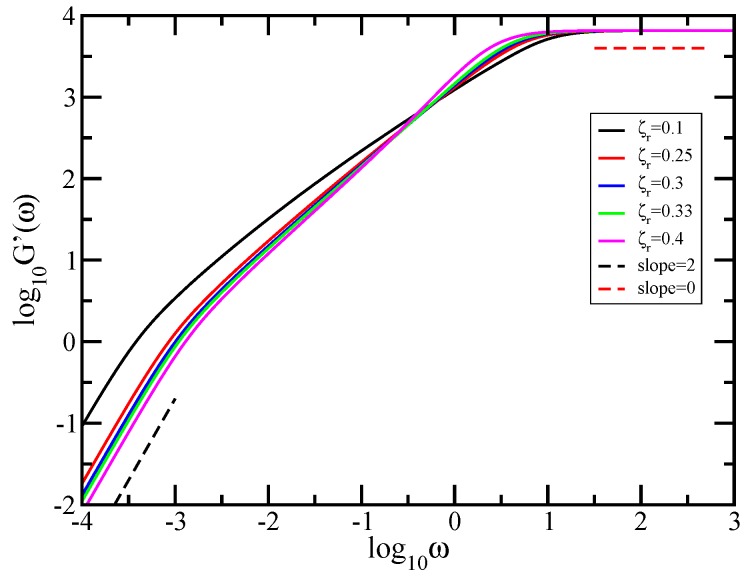
Storage modulus $G^{\prime }\left(\omega \right)$, displayed in dimensionless units for the fractal cactus network of size $N=3^{8}$ and hydrodynamic interaction strength $\zeta_{r}=0.1$, 0.25, 0.3, 0.33, and 0.4. For guidance, the dashed black line indicates slope 2 and the red dashed line indicates slope 0. Zimm model.

We continue the analysis with mechanical relaxation modulus $G^{\prime }\left(\omega \right)$, which we present in Figure 9. Here, we used a fractal cactus network consisting of $N=3^{8}$ monomers and the employed hydrodynamic interaction parameter extends from 0.1 to 0.4. Plotted are the results obtained by using Equation (8) in which we set σ=1 and C/N=1. In the same fashion as in Figure 7 and Figure 8, the curves obtained for several considered values of $\zeta_{r}$ are denoted with colors: black solid line ($\zeta_{r}=0.1$), red solid line ($\zeta_{r}=0.25$), blue solid line ($\zeta_{r}=0.3$), green solid line ($\zeta_{r}=0.33$), and magenta solid line ($\zeta_{r}=0.4$). As in the Rouse case, the limiting, connectivity-independent behaviors at very small frequencies $G^{\prime }\left(\omega \right)∼\omega^{2}$ and at very large frequencies $G^{\prime }\left(\omega \right)∼\omega^{0}$ are well rendered by the curves from the figure. This a common point for all generalized Gaussian structures. In the same manner as in Figure 5, as a guide to the eye, two reference (dashed) lines are plotted at the lower and at the upper limit; the dashed black line indicates slope 2 and the red dashed line indicates slope 0. Moreover, as before, the structure-dependent aspects are given by the intermediate regions. The first clear-cut feature is that, under HI, the intermediate region gets smaller than in the Rouse case. In the intermediate frequency domain, the storage modulus displays a very interesting behavior as a function of hydrodynamic interactions strength. For low and medium hydrodynamic interactions ($\zeta_{r}=0.1$ and 0.25), the curves in this in-between frequency region have a slightly concave shape, which in the double logarithmic scales of the figure means no scaling behavior. Instead, for slightly larger hydrodynamic interactions ($\zeta_{r}=0.3$ and 0.33), the curves appear as straight lines denoting power law behavior. The determined values of the slope are 0.988 for $\zeta_{r}=0.3$ and 1.023 for $\zeta_{r}=0.33$. Under stronger hydrodynamic interactions, the scaling is again lost. For $\zeta_{r}=0.4$, the curve in the intermediate frequency domain does not develop as a straight line, thus there is no signature of scaling in this region.

**Figure 10:**
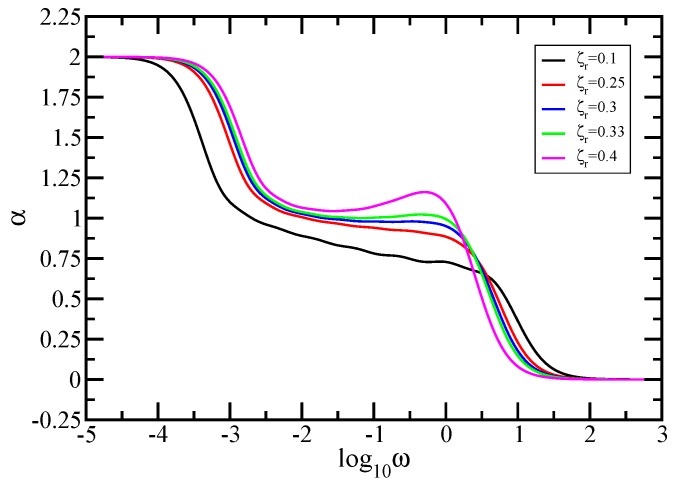
Local slopes α of the curves of Figure 9. The inset gives the considered values of the hydrodynamic interaction strength. Zimm model.

In the same manner as in Figure 8, we proceed to show in Figure 10 the quantity $\alpha =d(ln\;G^{\prime }\left(\omega \right))/d\left(ln\;\omega \right)$, the derivatives of the curves of Figure 9. Immediately apparent are, for very small and very large frequencies, the limiting theoretically expected values of the slope α, namely 2 and 0. Focusing now in the in-between region, we observe that the derivatives of the curves obtained under considering low and medium hydrodynamic interactions ($\zeta_{r}=0.1$ and 0.25) display no plateau, a sign that there is no scaling behavior. Instead of a plateau, we find a quite mild cross-over behavior. On the other hand, for slightly larger hydrodynamic interactions ($\zeta_{r}=0.3$ and 0.33), one sees clearly the appearance of the plateau that indicates scaling behavior. The determined plateau value for $\zeta_{r}=0.3$ is 0.99 and for $\zeta_{r}=0.33$ is 1.02. Under stronger hydrodynamic interactions ($\zeta_{r}=0.4$), the power law behavior is again lost, and the derivative does not show a plateau in the intermediate frequency region.

The eigenvalue spectrum of the product matrix is very sensitive to the modification of the hydrodynamic interaction strength. The obtained behaviors reflect the changes in the eigenvalue spectrum of the product matrix HA caused by the variation of the HI strength. For the same size *N*, an increase of the value of $\zeta_{r}$ leads to a significant lowering of the eigenvalues, especially the ones that have larger values. Because in the calculation of the storage modulus (Equation 8) the eigenvalues enter into the denominator, the increase of $\zeta_{r}$ leads to larger values of the storage modulus and thus to larger values of the local slope. In Figure 10, for the same frequency, the local slope in the intermediate frequency region increases with the increase of the value of $\zeta_{r}$. Moreover, for the same eigenvalue $\lambda_{i}$, the expression $\omega^{2}/\left(\omega^{2}+4\lambda_{i}^{2}\right)$ increases with the increase of the frequency ω, and it tends to 1 for $\omega \gg \lambda_{i}$. This produces a further increase in the value of the local slope. Therefore, for very large values of HI strength (as $\zeta_{r}=0.4$), almost all the eigenvalues have very low values and produce larger local slopes at higher intermediate frequencies, which together with the aforementioned increase leads to a maximum in the behavior of the local slope. For low and medium values of $\zeta_{r}$, the eigenvalue spectrum still contains sufficient larger eigenvalues on its upper part. Nevertheless, when comparing to curves ($\zeta_{r}=0.1$ and $\zeta_{r}=0.25$), the local slope does not increase uniformly (not equally) in the intermediate frequency region. There is a more pronounced increase from the medium to the larger intermediate frequencies than in the region of lower intermediate frequencies. For slightly larger values of $\zeta_{r}$, the faster increase in the region of medium to large intermediate frequencies equals the slower increase in the region of lower intermediate frequencies and one obtains a plateau.

In the Rouse case, the power law exponent depends on the spectral dimension. In the Zimm model, the scaling exponent of the storage modulus obtained under the consideration of slightly larger hydrodynamic interactions can be related to the value of the Gaussian fractal dimension. For the intermediate domain, a theoretical effective medium approach was developed by Cates [76] to support a scaling hypothesis for polymeric fractals; in this regime (called by him the high frequency regime), his approach suggests a power-law behavior for $G^{\prime }\left(\omega \right)$. Following this line of thought, the power-law exponent (slope of the curve in this region) should be

(33) $$\alpha_{T}=\frac{d_{fg}}{d},$$

where *d* represents the Euclidean dimension and it is equal to 3. Practically, the exponent $d_{s}/2$ from the Rouse case gets replaced in the Zimm case by $d_{fg}/d$. Now, from the comparison of the obtained slopes (especially that obtained for $\zeta_{r}=0.33$) with the theoretical value $\alpha_{T}=1.0566$, one observes a good agreement.

**Figure 11:**
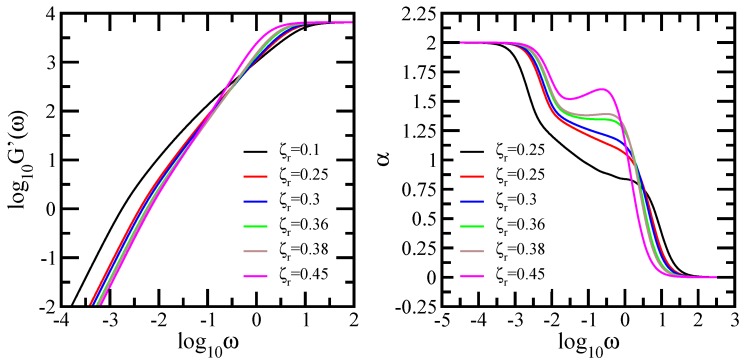
Left-hand side panel: Storage modulus $G^{\prime }\left(\omega \right)$, displayed in dimensionless units for the dual Sierpinski gasket with size $N=3^{8}$, and the hydrodynamic interaction strength $\zeta_{r}=0.1$, 0.25, 0.3, 0.36, 0.38, and 0.45. For guidance, the dashed black line indicates slope 2 and the red dashed line indicates slope 0. Right-hand side panel: Local slopes α of the curves of the left-hand side panel. Zimm model.

We note that the loss of scaling for the cases of low and medium HI strength parallel the findings of Ref. [25,39]), where similar non-scaling behavior in the Zimm-type approach for dual Sierpinski gaskets and Sierpinski hexagon was also reported. These works did not study the dynamics of the fractal networks under slightly larger or even stronger hydrodynamic interactions. We extend our study in the Zimm approach by investigating also the dynamical behavior of the storage modulus for the dual Sierpinski gaskets, insisting mostly on the cases of larger hydrodynamic interactions. The left-hand side panel of Figure 11 shows the behavior of the storage modulus of the dual Sierpinski gasket fractal at generation 8 (consequently $N=3^{8}$) under the influence of hydrodynamic interactions with strength ranging from 0.1 to 0.45. The storage modulus was calculated based on Equation (8), in which we set σ=1 and C/N=1, and it is presented in dimensionless units. The right-hand side panel of Figure 11 displays the quantity, $\alpha =d(ln\;G^{\prime }\left(\omega \right))/d\left(ln\;\omega \right)$, the derivatives of the curves of the left-hand side panel. We note that the scales of the left-hand side panel are double logarithmic to base 10, while in the right-hand side panel the *x*-axis is logarithmic to basis 10, and the *y*-axis is linear. The results obtained for each value of the HI strength are illustrated with color lines as follows: black solid line ($\zeta_{r}=0.1$), red solid line ($\zeta_{r}=0.25$), blue solid line ($\zeta_{r}=0.3$), green solid line ($\zeta_{r}=0.36$), brown solid line ($\zeta_{r}=0.38$), and magenta solid line ($\zeta_{r}=0.45$). As in Figure 9, for very high ω, we find $G^{\prime }\left(\omega \right)∼\omega^{0}$, which indicates a single-bead mechanical response, whereas, for very low frequencies, $G^{\prime }\left(\omega \right)∼\omega^{2}$, which represents the mechanical response of the entire fractal network. In the right-hand side panel, these regions correspond to α=0, respectively α=2. Focusing now in the intermediate frequency region, we observe no power law behavior for the cases of low and medium hydrodynamic interactions ($\zeta_{r}=0.1$ and 0.25), exactly as reported in the previous works [25,39]. Even for slightly larger hydrodynamic interactions, $\zeta_{r}=0.30$, the storage modulus does not obey power law behavior. These can be better seen in the derivatives, where no plateau is evident. Remarkably, the curves obtained for much larger HI strength (0.36 and 0.38) develop in the in-between region as straight lines and they indicate power law behavior. In the right-hand side panel, for each of these values of $\zeta_{r}$, we observe a very clear plateau. The best approximation to our data leads to α=1.35 for $\zeta_{r}=0.36$ and α=1.39 for $\zeta_{r}=0.38$, the last value being in good agreement with the theoretical value $\alpha_{T}=1.43377$, determined from Equation (33). The Gaussian fractal dimension of the dual Sierpinski gasket needed in Equation (33) was calculated from Equation (12) with spectral dimension $d_{s}=1.36521$ [39]. For much stronger hydrodynamic interactions ($\zeta_{r}=0.45$), the scaling behavior of the storage modulus is again lost. This is well rendered by the disappearance of the plateau in the derivative of the curve. The scaling argument of Equation (33) holds also for the Vicsek fractal. This loopless structure scales in the Zimm model. In Ref. [17], for Vicsek fractal with functionality f=3, the authors have reported for the scaling behavior of the storage modulus a power law exponent of 0.841. The spectral dimension of the Vicsek fractal (f=3) is $d_{s}=1.11577$. Inserting this value in Equation (12), we get $d_{fg}=2.52371$ and then, from Equation (33), we obtain α=0.841, exactly the value reported in Ref. [17].

The relaxation dynamics of the fractal cactus networks, studied in the Zimm model, shows a very strong dependence on the strength of the hydrodynamic interactions. Even though the structure contains only loops, under the influence of low and medium hydrodynamic interactions, the non-scaling behavior of the dynamical quantities is similar to that obtained for fractals that contain loops on loops. Comparing the dynamical behavior of the fractal cactus with that of dual Sierpinski gaskets, we observe that the presence of loops on loops practically maintains the non-scaling behavior for slightly large hydrodynamic interactions, shifting the scaling behavior towards higher hydrodynamic interaction strength. For the situations where the dynamical quantities obey power law, the power law exponent can be predicted by Equation (33). This formula turns out to fit also to other fractals.

## 5. Conclusions

In this work, we have studied the relaxation dynamics of a polymer network modeled by a fractal structure with a cactus-like shape. Our study has been performed in the framework of the GGS model by employing the Rouse and Zimm approaches. Additionally, in the Rouse-type approach, we have investigated the behavior of the mean-square radius of gyration. In order to be able to treat the dynamics of very large structures, based on real-space renormalization transformations, we have developed an iterative method for the determining of the whole eigenvalue spectrum of the connectivity matrix. With the eigenvalues achieved in the iterative manner, we have calculated the relaxation quantities for very large generations of the fractal network.

The general picture that emerges in the Rouse-type approach is that the dynamical quantities obey power law behavior and the sole parameter of importance for the relaxation dynamics is the spectral dimension. In addition, in the Rouse model, based on the recursive polynomial and using Vieta’s formulas, we have derived a very useful analytical expression for the determining of the mean-square radius of gyration of the fractal cactus network. Only the size of the fractal is needed in order to calculate the mean-square radius of gyration. Furthermore, we have inferred a simple analytical form which highlights the scaling dependence of $R_{g}^{2}$ on the spectral dimension.

The introduction of hydrodynamic interactions, on the other hand, vastly changes the Rouse picture. Our use of the Zimm formalism, based on the preaveraged Oseen tensor, leads to the conclusion that the behavior of relaxation quantities shows a very strong dependence on the strength of the hydrodynamic interactions. For low and medium hydrodynamic interactions, the dynamical quantities do not obey power law behavior. For slightly larger hydrodynamic interactions, the relaxation dynamics of the fractal network is governed by power law. Under strong hydrodynamic interactions, the storage modulus does not follow power law behavior and the average displacement of the monomer is very low. For the storage modulus, following the scaling argument of Cates, we have shown that the theoretical expected power law exponent relates to the Gaussian fractal dimension. Moreover, under the consideration of large hydrodynamic interactions, we have highlighted a scaling behavior for the storage modulus of the dual Sierpinski gasket. Impressively, the power law exponent predicted by Equation (33) fits properly for the dual Sierpinski gasket and also for the Vicsek fractal.

Beside the relaxation quantities studied in this paper, many other dynamical processes can be investigated by making use of the connectivity matrix—for instance, mean first passage time of a random walk [16], the dielectric relaxation functions [17], the NMR relaxation functions [29,66,84], and quantum transport in complex networks [85,86]. Therefore, the knowledge of the eigenvalue spectrum of the matricial form describing the connectivity of the polymer is of great importance, leading to further interdisciplinary scientific advances.

We have compared our general theoretical features found for the fractal cactus network with experimental results from the literature. Remarkably, our theoretical findings in the Rouse model are well supported by mechanical relaxation experiments performed on different types of polymers. We address the fractal cactus network as a possible theoretical model for the relaxation dynamics of different polymer systems as cross-linked polymer networks, micelle networks, and polymer gels.

## Figures and Tables

**Figure A1:**
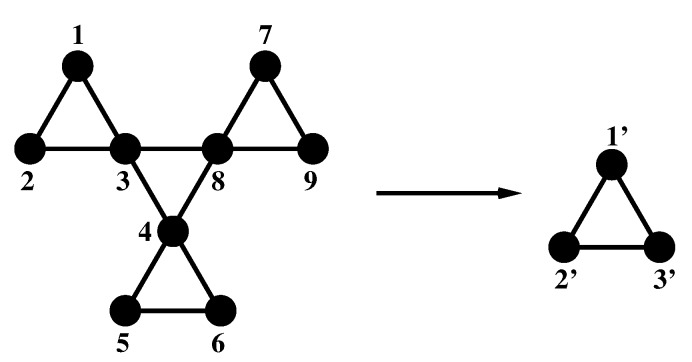
First transformation.

**Figure A2:**
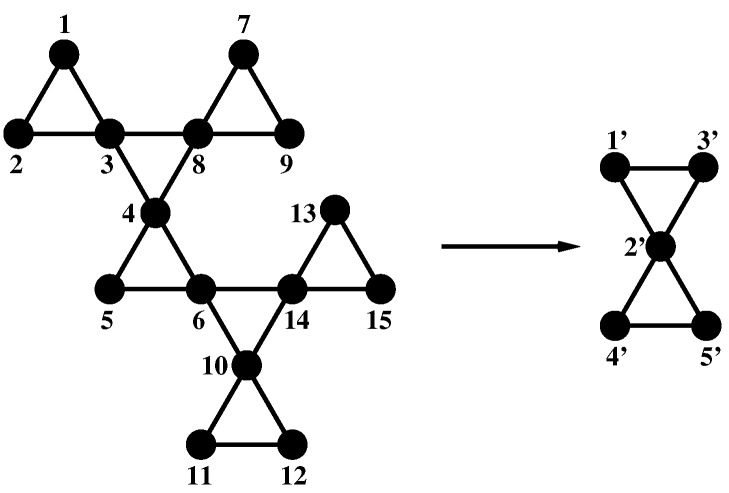
Second transformation.

## Abbreviations

g	generation of the fractal cactus
HI	hydrodynamic interaction
$\zeta_{r}$	hydrodynamic interaction strength (or parameter)
GGS	generalized Gaussian structures

## Appendix A. Decimation Procedure

Here, we present the details of the decimation procedure outlined in Section 3. The decimation procedure relies on two real-space renormalization transformations under which the fractal cactus network reduces from generation *g* to generation g−1. The first transformation is sketched in Figure A1. Our starting point is Equation (13), which gives the relations between the components $\phi_{k}$ of the eigenvector Φ belonging to a certain eigenvalue $\lambda_{k}$. The main eigenvalue equations which we need for our calculations are:

(A1) $$\left(2-\lambda \right)\phi_{1}=\phi_{2}+\phi_{3},$$

(A2) $$\left(2-\lambda \right)\phi_{2}=\phi_{1}+\phi_{3},$$

(A3) $$\left(4-\lambda \right)\phi_{3}=\phi_{1}+\phi_{2}+\phi_{4}+\phi_{8},$$

(A4) $$\left(4-\lambda \right)\phi_{4}=\phi_{3}+\phi_{5}+\phi_{6}+\phi_{8},$$

(A5) $$\left(4-\lambda \right)\phi_{8}=\phi_{3}+\phi_{4}+\phi_{7}+\phi_{9}.$$

Equations (A1) and (A3) can be combined, leading to

(A6) $$\left[2-\left(-\lambda^{2}+6\lambda \right)\right]\phi_{1}=-4\phi_{1}+3\phi_{2}+\phi_{3}+\phi_{4}+\phi_{8}.$$

By inserting the expression of $\phi_{3}$ from Equation (A3) into Equation (A2), one gets

(A7) $$\left[2-\left(-\lambda^{2}+6\lambda \right)\right]\phi_{2}=3\phi_{1}-4\phi_{2}+\phi_{3}+\phi_{4}+\phi_{8}.$$

Introducing the expressions for $\phi_{1}$ and $\phi_{2}$ given by Equations (A1) and (A2) into Equation (A3) yields

(A8) $$\left[2-\left(-\lambda^{2}+6\lambda \right)\right]\phi_{3}=\phi_{1}+\phi_{2}-2\phi_{3}-\phi_{4}+\phi_{5}+\phi_{6}+\phi_{7}-\phi_{8}+\phi_{9}.$$

Summing up Equations (A6), (A7), and (A8) and then making use of few algebraic calculations, one obtains

(A9) $$\left[2-\left(-\lambda^{2}+6\lambda \right)\right]\left(\phi_{1}+\phi_{2}+\phi_{3}\right)=\phi_{4}+\phi_{5}+\phi_{6}+\phi_{7}+\phi_{8}+\phi_{9}.$$

Now, by setting in Equation (A9),

(A10) $$P\left(\lambda \right)=-\lambda^{2}+6\lambda ,$$

(A11) $$\psi_{1^{\prime }}=\phi_{1}+\phi_{2}+\phi_{3},$$

(A12) $$\psi_{2^{\prime }}=\phi_{4}+\phi_{5}+\phi_{6},$$

(A13) $$\psi_{3^{\prime }}=\phi_{7}+\phi_{8}+\phi_{9},$$

we get

(A14) $$\left[2-P\left(\lambda \right)\right]\psi_{1^{\prime }}=\psi_{2^{\prime }}+\psi_{3^{\prime }},$$

which is exactly Equation (17) from the main text.

The second transformation is displayed in Figure A2. Our starting point is again Equation (13). The main eigenvalue equations which we use in our calculations are:

(A15) $$\left(4-\lambda \right)\phi_{3}=\phi_{1}+\phi_{2}+\phi_{4}+\phi_{8},$$

(A16) $$\left(4-\lambda \right)\phi_{4}=\phi_{3}+\phi_{5}+\phi_{6}+\phi_{8},$$

(A17) $$\left(2-\lambda \right)\phi_{5}=\phi_{4}+\phi_{6},$$

(A18) $$\left(4-\lambda \right)\phi_{6}=\phi_{4}+\phi_{5}+\phi_{10}+\phi_{14},$$

(A19) $$\left(4-\lambda \right)\phi_{8}=\phi_{3}+\phi_{4}+\phi_{7}+\phi_{9},$$

(A20) $$\left(4-\lambda \right)\phi_{10}=\phi_{6}+\phi_{11}+\phi_{12}+\phi_{14},$$

(A21) $$\left(4-\lambda \right)\phi_{14}=\phi_{6}+\phi_{10}+\phi_{13}+\phi_{15}.$$

Inserting Equation (A15) and (A19) into Equation (A16) and after performing some simple algebraic calculations leads to

(A22) $$\left[4-\left(-\lambda^{2}+6\lambda \right)\right]\phi_{4}=\phi_{1}+\phi_{2}-\phi_{3}+\phi_{5}-\phi_{6}+\phi_{7}-\phi_{8}+\phi_{9}+\phi_{10}+\phi_{14}.$$

With the expression of $\phi_{4}$ from Equation (A16), Equation (A17) becomes

(A23) $$\left[4-\left(-\lambda^{2}+6\lambda \right)\right]\phi_{5}=\phi_{3}+\phi_{4}-2\phi_{5}+\phi_{6}+\phi_{8}+\phi_{10}+\phi_{14}.$$

In the same fashion as above, by inserting the forms of $\phi_{10}$ and $\phi_{14}$ given by Equations (A20) and (A21) into Equation (A18), one has

(A24) $$\left[4-\left(-\lambda^{2}+6\lambda \right)\right]\phi_{6}=\phi_{3}-\phi_{4}+\phi_{5}+\phi_{8}-\phi_{10}+\phi_{11}+\phi_{12}+\phi_{13}-\phi_{14}+\phi_{15}.$$

Summing up Equations (A22)–(A24) and after few algebraic calculations and rearranging terms, one obtains

(A25) $$\left[4-\left(-\lambda^{2}+6\lambda \right)\right]\left(\phi_{4}+\phi_{5}+\phi_{6}\right)=\phi_{1}+\phi_{2}+\phi_{3}+\phi_{7}+\phi_{8}+\phi_{9}+\phi_{10}+\phi_{11}+\phi_{12}+\phi_{13}+\phi_{14}+\phi_{15}.$$

Now, by setting in Equation (A25)

(A26) $$P\left(\lambda \right)=-\lambda^{2}+6\lambda ,$$

(A27) $$\psi_{1^{\prime }}=\phi_{1}+\phi_{2}+\phi_{3},$$

(A28) $$\psi_{2^{\prime }}=\phi_{4}+\phi_{5}+\phi_{6},$$

(A29) $$\psi_{3^{\prime }}=\phi_{7}+\phi_{8}+\phi_{9},$$

(A30) $$\psi_{4^{\prime }}=\phi_{10}+\phi_{11}+\phi_{12},$$

(A31) $$\psi_{5^{\prime }}=\phi_{13}+\phi_{14}+\phi_{15},$$

we obtain

(A32) $$\left[4-P\left(\lambda \right)\right]\psi_{2^{\prime }}=\psi_{1^{\prime }}+\psi_{3^{\prime }}+\psi_{4^{\prime }}+\psi_{5^{\prime }},$$

which is exactly Equation (16) from the main text.
